# Network analysis of emotional symptoms and cognitive function changes in primary insomnia

**DOI:** 10.3389/fpsyg.2025.1520448

**Published:** 2025-07-21

**Authors:** Ling Xu, Wantao Ding, Hui Guo

**Affiliations:** ^1^Department of Clinical Psychology, Wenzhou Seventh People’s Hospital, Wenzhou, China; ^2^Department of Psychiatric Rehabilitation, Wenzhou Seventh People’s Hospital, Wenzhou, China

**Keywords:** primary insomnia, emotional symptoms, cognitive dysfunction, anxiety, depression

## Abstract

**Background:**

Primary insomnia is a common and complex disorder characterized by a range of symptoms and consequences of varying severity. Long-term sleep disturbances can lead to anxiety, depression and cognitive dysfunctions. This study aimed to investigate the relationship between emotional changes and cognitive dysfunction in patients with primary insomnia and to explore the factors influencing the disorder.

**Methods:**

The study participants included 40 patients with primary insomnia diagnosed by the International Classification of Sleep Disorders-3 and 48 healthy controls from the Seventh People’s Hospital of Wenzhou. The Pittsburgh Sleep Quality Index, Montreal Cognitive Assessment, Hamilton Anxiety Scale and Hamilton Depression Scale were used to assess clinical symptoms. *T*-tests, chi-square tests, Pearson correlation and network analysis were used to examine the mutual relationship between emotional symptoms and cognitive function in participants with primary insomnia.

**Results:**

Both immediate and delayed memory of the primary insomnia group was significantly worse than that of the healthy controls group, whereas there was no significant difference in long-term delayed recognition between the two groups. The naming and language scores of the primary insomnia group were significantly lower than those of the healthy controls group. The total Pittsburgh Sleep Quality Index score was positively correlated with anxiety and depression symptoms.

**Conclusion:**

This study emphasized the correlation between emotional symptoms and cognitive dysfunction in patients with primary insomnia. Simultaneously, their levels of anxiety and depression were both positively correlated with the degree of cognitive dysfunction.

## Highlights

- Network analysis revealed bidirectional relationships between insomnia symptoms, emotional symptoms and cognitive functioning.
- Insomnia symptoms led to a decline in both immediate and delayed memory, while long-term recognition was not affected.
- In primary insomnia, there was a notable reduction in naming and language proficiency scores.

## Introduction

Given the rising prevalence of insomnia, currently standing at 27%, the attendant risks have become more apparent. There is a general consensus within the scientific community that chronic insomnia significantly and adversely affects an individual’s cognitive functioning and emotional health. Research has shown that patients with primary insomnia generally suffer from impaired cognitive functions, such as reduced memory, impaired attention and decreased executive function (Ballesio, 2017; Lu et al., 2023; Subbotina and Poluektov, 2015; Xu et al., 2011). Additionally, researchers have explored the bidirectional relationship between insomnia, anxiety and depression through cross-sectional and longitudinal research methods, finding that long-term insomnia not only aggravates depressive and anxious symptoms, but also that depressive and anxious symptoms can in turn negatively affect sleep quality, forming an intensive cycle (Swanson and Pickett, 2010; Pickett et al., 2010). In intervention studies, a combination of cognitive behavioral therapy and pharmacotherapy has shown some effectiveness in improving sleep and the accompanying anxiety and depressive symptoms (Jin et al., 2021; Godzik et al., 2021). Several large cohort studies in Europe and North America have revealed that patients with primary insomnia show structural and functional connectivity changes in the brain, especially abnormalities in the prefrontal cortex, hippocampus and amygdala, which are closely related to cognitive function impairment and mood disorders (Xu et al., 2023). Additionally, international research has focused on the role of biomarkers (such as inflammatory factors in the blood and neurotransmitter levels) in the co-occurrence of insomnia and depression or anxiety, thus providing a new perspective for treatment (Lin and Xu, 2023; He et al., 2021).

The complex interaction mechanisms have not been fully elucidated despite the recognition in existing studies of significant correlations between primary insomnia and cognitive dysfunction, anxiety and depression. In particular, how insomnia-induced neurotransmitter imbalance and brain region functional and structural changes directly affect cognitive function and emotion regulation, as well as the details of the interaction mechanism between these processes, which still require considerable investigation (Su et al., 2024; Li et al., 2024). In particular, there is a lack of systematic studies on the dynamic changes in cognitive decline speed, anxiety and depressive symptoms and their mutual interaction within insomnia patients over time. Although cognitive behavioral therapy and pharmacotherapy are widely used in various mental disorders (Meaklim et al., 2024; Quin et al., 2024; Mak et al., 2024; Loughan et al., 2024; Li et al., 2024; Lee et al., 2024; Furukawa et al., 2024; Feng et al., 2024), there is not enough research on the comprehensive effects of the integrated intervention for insomnia-accompanied cognitive dysfunction and anxiety/depression, especially in regard to long-term effects, side effects, recurrence rate and patient satisfaction. Cognitive behavioral therapy requires the guidance of seasoned therapists or clinicians and demands a certain level of self-discipline from patients (Youngren et al., 2023; Vargas, 2023). Sleep quality may not show improvement and potentially worsens during the initial couple of weeks of cognitive behavioral therapy implementation. For insomnia patients with comorbid anxiety or depression, adherence is suboptimal, resulting in lower satisfaction than anticipated (Maruani et al., 2023). Conversely, this can lead to decreased patient trust and impede treatment progress. The possible risks of drug treatment must also be considered. Long-term use of hypnotics, will produce drug dependence, if a drug is suddenly withdrawn, it can cause rebound insomnia more serious than the insomnia before medication.

To investigate the interactions among various factors, we employed a network analysis model. The mental network analysis goes beyond traditional analysis by conceptualizing mental health issues as a complex system of interacting symptoms. It maps symptoms as nodes and their relationships as edges, allowing for precise analysis of the symptom network. Centrality measures help identify key symptoms for intervention and prevention. This approach deepens our understanding of mental health challenges and reduces risks of complications. This study aimed to comprehensively investigate the relationship between cognitive dysfunction and anxiety and depressive symptoms in patients with primary insomnia through an interdisciplinary approach. By integrating the biopsychosocial dimension, this research systematically elucidates the complex interactive mechanisms between sleep disorders and cognitive and emotional disturbances, thereby providing more precise diagnostic strategies, intervention approaches, and treatment pathways for clinical practice. The study is expected to provide in depth evidence on the complex association between cognitive dysfunction, anxiety and depressive symptoms in patients with primary insomnia and to promote the development of precision intervention strategies.

## Methods

### Study design and participants

This study investigated outpatients receiving clinical care in Wenzhou Seventh People’s Hospital of China. The enrollment period ranged from December 2020 to January 2022. The inclusion criteria were: (1) Aged between 18 and 60 years; (2) Diagnosed with primary insomnia; (3) Residing in Wenzhou; (4) Provision of informed consent. Exclusion criteria included: (1) Previous history of major mental illness or neurological disorders; (2) Intellectual disability; (3) Dyslexia or cognitive impairment; (4) Regular use of sedative sleep aid within 3 months. Healthy controls were recruited from outpatients who had not been diagnosed with insomnia, anxiety or depression after initial screening. To ensure comparability with the insomnia group, the controls were matched on age, gender and educational years. Age: The age range of the insomnia group was determined, and controls were recruited within a ± 5-year age range of the mean age of the insomnia group. Gender: The gender distribution in the insomnia group was noted, and controls were selected to have a similar gender ratio. Educational years: The education level of the insomnia participants was assessed, and controls were matched to have a comparable level of education. Consequently, the final sample comprised 88 participants. All participants were assessed for sleep, mood and cognitive function including PSQI, HAMA, HAMD and MoCA scales. The clinical scale evaluation and neuropsychological tests involved in this project were performed by skilled medical researchers. The study was carried out in accordance with the recommendations of the research ethics committee of Wenzhou Seventh People’s Hospital with written informed consent from all participants.

### Measurements

#### Demographic information

Demographic information of the patients diagnosed with primary insomnia by International Classification of Sleep Disorders-3 (ICSD-3) was extracted from the outpatient system. In the healthy control group, demographic data were collected using a self-compiled questionnaire. Demographic variables included sex, age and years of education. Gender was dummy coded as a dichotomous variable, with 0 denoting male and 1 denoting female. Age and years of education were treated as continuous variables and measured in years.

#### Insomnia

Sleep quality in all participants was assessed using the Pittsburgh Sleep Quality Index (PSQI). PSQI was used to assess the sleep quality of the participants in the last month. It is composed of 19 self-rated items and five other-rated items, of which the 19 self-rated items and 5 other-rated items do not participate in the scoring. The total score of each component was the PSQI total score. The total score ranged from 0 to 21 and the higher the score, the worse the sleep quality. The internal consistency coefficient of the scale was 0.84 (Buysse et al., 1989).

#### Cognition

Cognitive function was assessed using the Montreal Cognitive Assessment (MoCA) (Lau et al., 2024). Participants completed tasks such as trial making, serial subtraction, delayed recall of words, verbal fluency and abstraction. Scores on individual tasks were summed to obtain a total score, with higher scores indicating better cognitive functioning. The MoCA has demonstrated good sensitivity and specificity in detecting cognitive dysfunction across different populations, making it suitable for assessing cognitive function in clinical and research settings (Cronbach’s *α* = 0.86) (Nasreddine et al., 2005).

#### Anxiety

Anxiety levels were assessed using the Hamilton Anxiety Rating Scale (HAMA) (Gjerris et al., 1983). HAMA consists of 14 items covering both psychological and somatic symptoms of anxiety, such as tension, apprehension, nervousness and insomnia. Each item is rated on a scale from 0 to 4, with higher scores indicating a greater severity of anxiety symptoms. HAMA is widely used in clinical practice and research studies, with a Cronbach’s *α* of 0.86 (Maier et al., 1988).

#### Depression

Depressive symptoms were assessed using the Hamilton Depression Rating Scale (HAMD) (Maier, 1990). The HAMD scale consists of 21 items, each of which is rated on a scale from 0 to 4 or 0 to 2, with higher scores indicating a greater severity of depressive symptoms. It provides a reliable and valid measure of depressive symptom severity and is widely used in clinical practice and research studies to assess treatment responses and track changes in depressive symptoms over time (Zheng et al., 1988).

### Statistical analyses

Initially, descriptive statistics were calculated for the demographic variables, clinical measures and cognitive outcomes. Independent sample *t*-tests were used to compare the means of continuous variables between the healthy control and primary insomnia groups, while chi-square tests were employed for categorical variables. Independent sample *t*-tests were conducted to investigate the differences in PSQI factors and MoCA subscales between the two groups with the measurement data conforms to the normal distribution. Pearson’s correlation coefficients were computed to assess the associations between the PSQI total score and cognitive/emotional variables that showed significant between-group differences.

The sample size was estimated based on the parameters of an independent samples *t*-test. A large effect size (Cohen’s *d* = 0.8), was assumed as per Cohen’s convention (Cohen, 1988). The significance level (*α*) was set at 0.05, and the desired statistical power (1−*β*) was 0.8, which is commonly used in behavioral and medical sciences. Using these parameters, the sample size was calculated using the formula: *n* ≈ (4/*d*^2^) * (Z*α*/2 + Z1−*β*)^2^, where: *d* is the effect size (0.8) Z*α*/2 is the critical value of the normal distribution at *α*/2 (1.96) Z1−*β* is the critical value of the normal distribution at *β* (0.84). This calculation yielded a minimum estimated sample size of approximately 26 participants per group, or a total of 52 participants. A total of 88 participants were included in this study, to meet the requirements of statistical testing.

Subsequently, a network analysis was performed to explore the complex relationships between the PSQI factors and cognitive/emotional variables. The network was estimated using a Gaussian graphical model with graphical lasso regularization (Friedman et al., 2008). The extended Bayesian information criterion was used for model selection and the tuning parameter was set to 0.5, to ensure a balance between sparsity and sensitivity. The network structure was visualized using the qgraph package, with nodes representing the variables and edges representing partial correlations between them. The strength of the edges were determined by their absolute weights, with thicker edges indicating stronger associations.

Data were analyzed using SPSS version 26.00 and R version 4.2.3, statistical significance was set at *p* < 0.05 for all analyses.

## Results

### Descriptive analysis

Demographic characteristics and clinical measures of anxiety and depression in the healthy control (*n* = 48) and primary insomnia (*n* = 40) groups are presented in Table 1. No significant differences were found between the control and test groups in terms of sex, age, or educational level. The primary insomnia group reported significantly higher levels of anxiety (*t* = 14.54, *p* < 0.001) and depression (*t* = 12.65, *p* < 0.001) than did the healthy control group. Table 1 gives the significant differences in the PSQI factors between the healthy control and primary insomnia groups. The primary insomnia group scored significantly higher on all PSQI factors including sleep quality, sleep latency, sleep duration, sleep efficiency, sleep disturbances, use of sleep medication and daytime dysfunction (*p* < 0.001).

**Table 1:** Comparative analysis between healthy control and primary insomnia groups.

Variables	Health (*n* = 48)	Insomnia (*n* = 40)	χ^2^/t	*p*
Gender			1.65	0.20
Male	13 (27.08%)	9 (22.50%)		
Female	35 (72.92%)	31 (77.50%)		
Age	39.81 ± 9.93	37.28 ± 6.07	1.54	0.23
Educational years	9.27 ± 4.45	10.75 ± 3.51	1.64	0.10
Anxiety	3.25 ± 2.42	13.90 ± 4.39	14.54	<0.001
Depression	3.23 ± 2.90	14.83 ± 5.61	12.65	<0.001
PSQI	3.13 ± 1.70	14.51 ± 3.29	20.78	<0.001
Sleep quality	0.55 ± 0.50	2.46 ± 0.60	16.32	<0.001
Sleep latency	0.66 ± 0.70	2.24 ± 0.99	8.72	<0.001
Sleep duration	0.17 ± 0.38	2.46 ± 0.90	15.97	<0.001
Sleep efficiency	0.26 ± 0.61	2.63 ± 0.70	17.10	<0.001
Sleep disturbances	0.89 ± 0.43	1.41 ± 0.55	5.00	<0.001
Use of sleep medication	0.04 ± 0.20	1.66 ± 1.32	8.31	<0.001
Daytime dysfunction	0.55 ± 0.69	1.61 ± 1.02	5.76	<0.001

**Figure 1:**
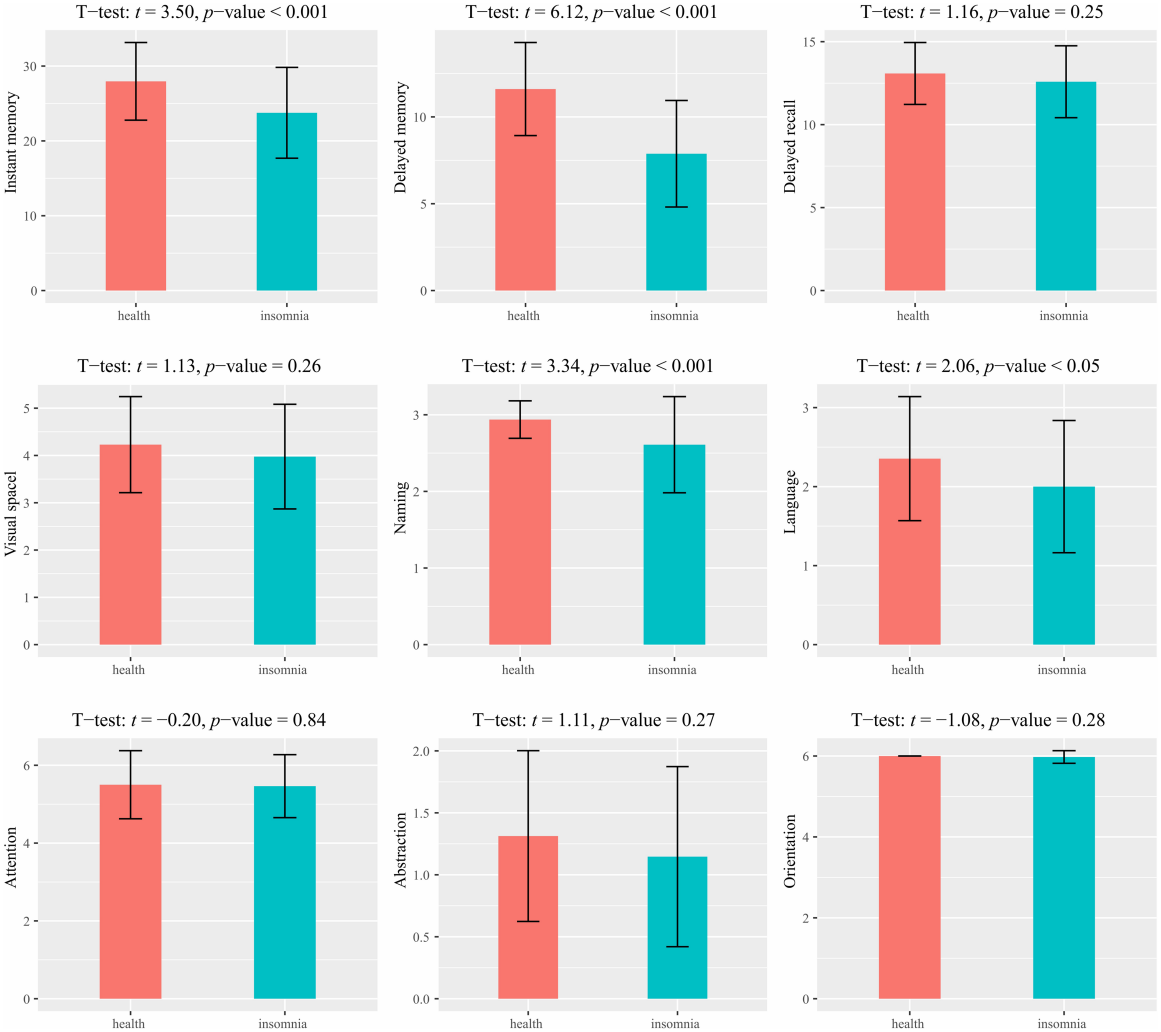
Comparison of memory and cognition between healthy controls and primary insomnia groups.

A group comparison of the MoCA results is shown in Figure 1. Significant differences were observed between the healthy control and primary insomnia groups in immediate memory (*t* = 3.50, *p* < 0.001) and delayed memory (*t* = 6.12, *p* < 0.001); the primary insomnia group performed worse in both domains. No significant difference was found in the long-delayed recognition\between the two groups. Regarding MoCA, significant between-group differences were found in naming (*t* = 3.34, *p* < 0.001) and language (*t* = 2.06, *p* < 0.05), with the primary insomnia group scoring lower on both domains. No significant differences were observed in other cognitive executive functions.

### Associations between sleep and emotional/cognitive functioning

**Figure 2:**
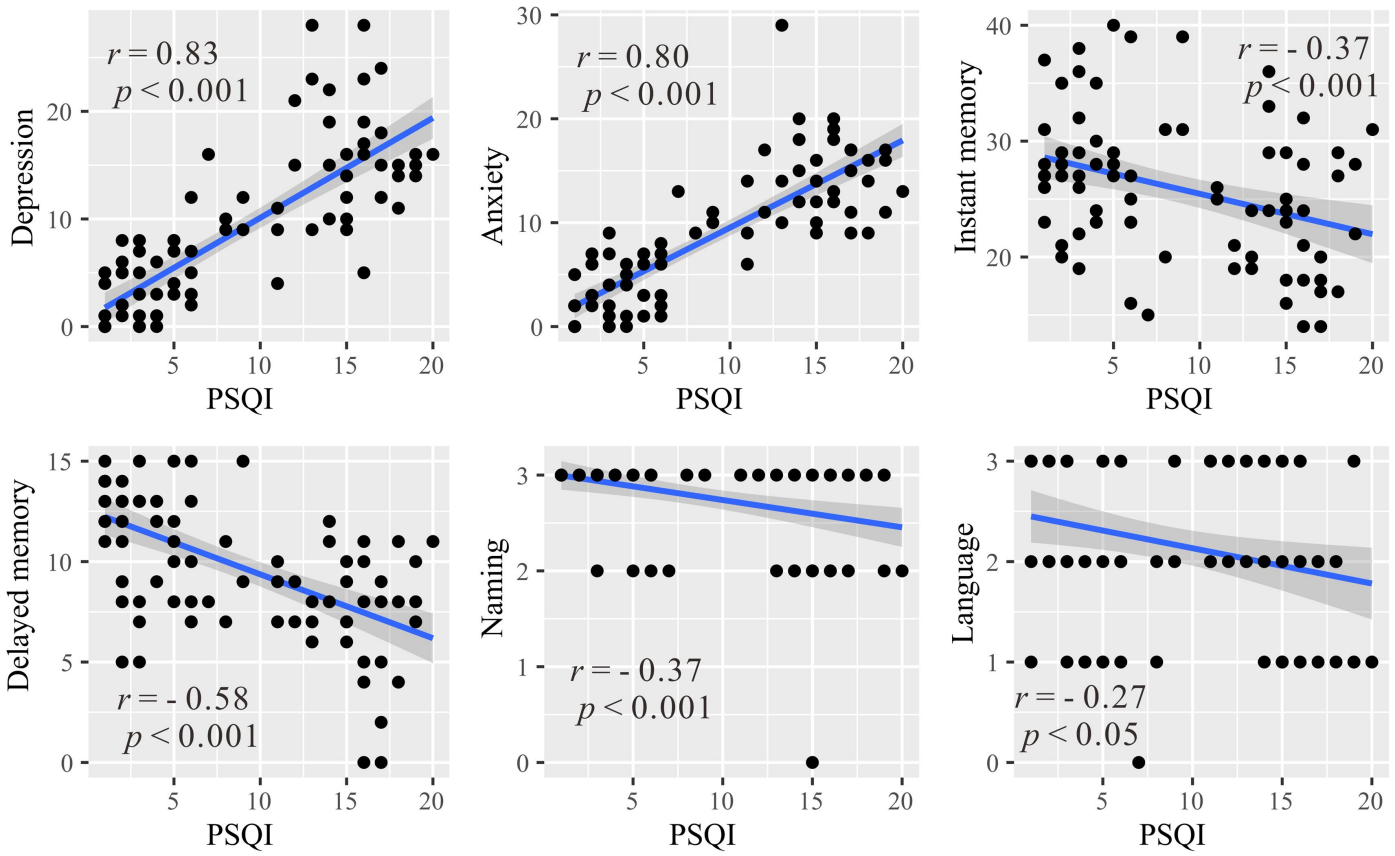
Correlations between the Pittsburgh Sleep Quality Index and emotional/cognitive variables with significant between-group differences (anxiety, depression, immediate memory, delayed memory, naming and language). *r* represents the Pearson correlation coefficient, and *p* denotes the statistical significance level.

To investigate the relationship between sleep and emotional/cognitive variables that showed significant differences between the healthy control and primary insomnia groups (i.e., anxiety, depression, immediate memory, delayed memory, naming and language), correlation analysis was conducted between these variables and the PSQI score (Figure 2). Significant positive correlations were found between the PSQI total score and anxiety (*r* = 0.83, *p* < 0.001) and depression (*r* = 0.80, *p* < 0.001), indicating that higher levels of sleep disturbance were associated with increased anxiety and depression symptoms. Instant memory (*r* = −0.58, *p* < 0.001) and delayed memory (*r* = −0.37, *p* < 0.001) were significantly negatively correlated with the PSQI score, suggesting that individuals with more severe sleep disturbances performed worse in these episodic memory domains. Similarly, naming (*r* = −0.37, *p* < 0.001) and language (*r* = −0.27, *p* = 0.02) were negatively correlated with the PSQI total score, indicating that higher levels of sleep disturbances were associated with lower scores in these cognitive domains.

### Network analysis of sleep and cognitive/emotional variables

**Figure 3:**
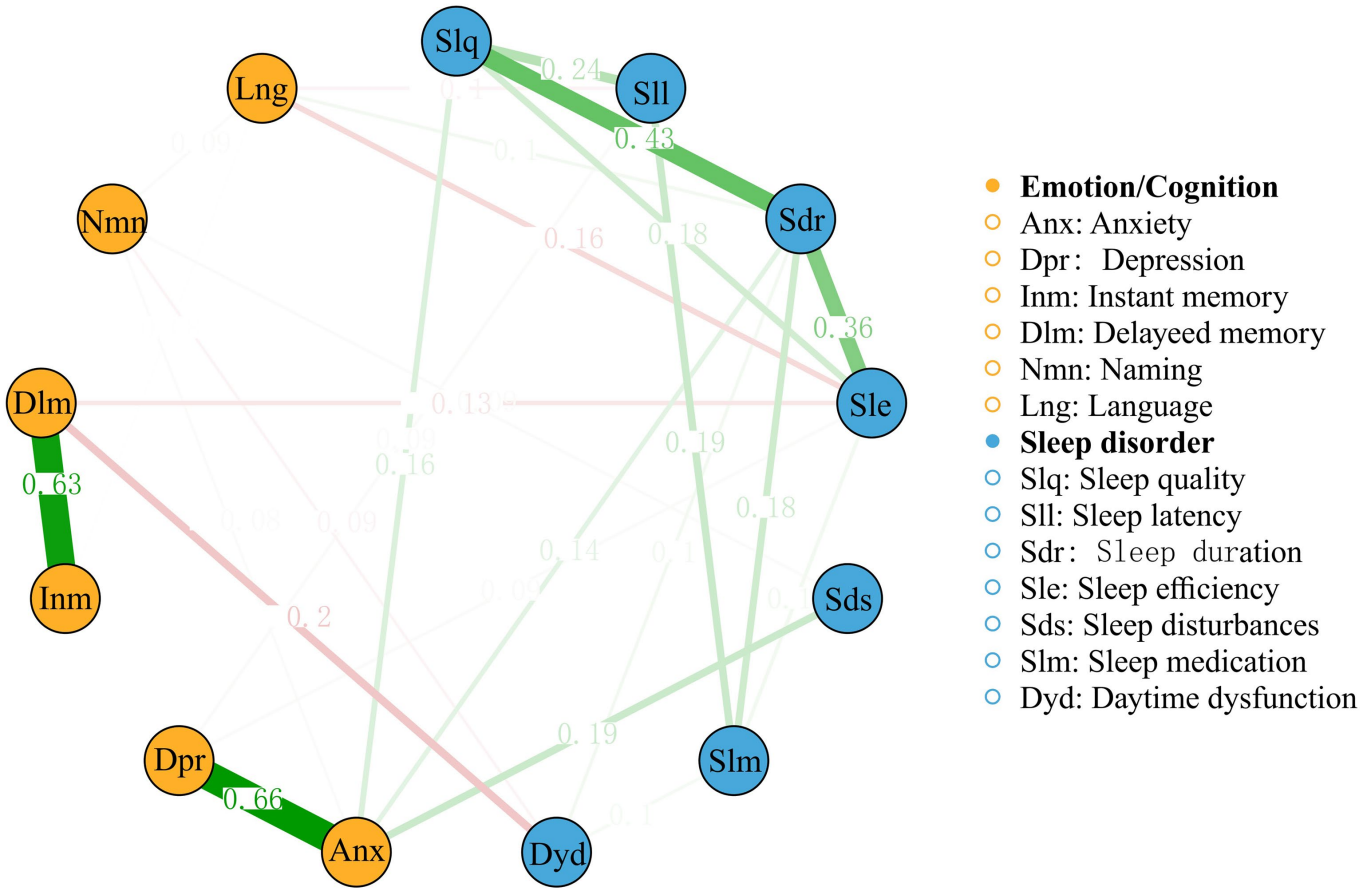
A network analysis of relationships among the Pittsburgh Sleep Quality Index factors and the cognitive/emotional variables.

To further explore the complex relationships among the PSQI factors and cognitive/emotional variables that showed significant between-group differences, a network analysis was performed (see Figure 3). The network had 46 non-zero edges out of a possible 78 (58.97%), with a mean weight of 0.04.

The network analysis revealed several notable connections. The strongest edge connection between these two categories was observed between delayed memory and daytime dysfunction, with an edge weight of −0.20. Anxiety and depression appear to be strongly connected to each other and to various sleep-related factors, such as sleep quality, sleep latency, and daytime dysfunction. The strong associations between these variables highlight the bidirectional relationship between sleep and emotional well-being, where poor sleep quality may exacerbate anxiety and depression symptoms, whereas emotional disturbances may contribute to the development and maintenance of primary insomnia. Furthermore, network analysis showed connections between PSQI factors and cognitive variables. Sleep quality and sleep efficiency are connected to immediate memory and delayed memory, suggesting that poor sleep quality and reduced sleep efficiency may have a detrimental impact on episodic memory functioning. Additionally, sleep disturbances and daytime dysfunction are connected to naming and language, indicating that disrupted sleep and daytime impairments may affect cognitive domains related to verbal abilities.

## Discussion

This study explored the correlation between cognitive function impairment and anxiety and depression symptoms in patients with primary insomnia to reveal the underlying mechanism of their interconnection and influencing factors and to provide theoretical and practical guidance for clinical intervention. These findings contribute to a better understanding of the dynamic nature of primary insomnia and provide valuable insights into clinical practice. The main findings and discussion points are as follows.

The research data strongly support that patients with primary insomnia have significant cognitive function impairment, especially in memory, language and naming, which is significantly different from that of the control group. This emphasizes the negative impact of long-term insomnia on cognitive function and highlights the importance of improving sleep quality for cognitive maintenance and promotion, which aligns with previous studies (Omer, 2023). Additionally, cognitive dysfunction induced by sleep primarily affects the delayed memory function. Previous research has demonstrated the crucial role of sleep in memory consolidation, particularly in the deep (slow-wave) and rapid eye movement stages (Cheng and Werning, 2013; Casey et al., 2016). During these stages, the brain reactivates and strengthens the neural connections formed during learning throughout the day, facilitating the conversion of short-term memories into long-term memories. Insomnia, particularly chronic insomnia, diminishes the duration of these critical sleep stages and disrupts the memory consolidation process, leading to a decline in delayed memory capacity. These findings were consistent with the results of the present study.

Results showed that insomnia was closely related to anxiety and depression in patients with primary insomnia. This observation is consistent with previous research that showed that insomnia, anxiety and depression are closely interconnected and mutually reinforcing (Xiao et al., 2023). Clinical interventions should consider the interconnection between insomnia, anxiety and depression and provide comprehensive treatment for patients with primary insomnia. Consequently, clinical interventions should transcend the traditional approach of treating each condition separately. This model should addresses not only the sleep disturbances associated with insomnia but also the underlying emotional and psychological factors of anxiety and depression. By doing so, we can effectively target the root causes of the patient’s symptoms, improve their overall quality of life, and enhance the long-term prognosis of their mental and physical health. This study further confirmed the bidirectional influence between insomnia and symptoms of anxiety and depression, that is, insomnia aggravates emotional disorders, while emotional problems also affect sleep quality (Kang et al., 2020). Lack of adequate sleep can affect emotional regulation and make individuals more likely to feel anxious or depressed. Insomnia may interfere with normal biological rhythms and affect levels of neurotransmitters in the brain, such as serotonin and dopamine, changes that have been linked to anxiety and depression. Fatigue and poor concentration due to insomnia can affect the quality of daily life, which in turn can produce negative emotional experiences. People with anxiety and depression often experience sleep disturbances, including difficulty falling asleep, waking at night, and waking up early. Anxiety and depression may cause the body to be in a constant state of stress, increasing cortisol levels and interfering with normal sleep cycles. Worry and negative thought cycles can interfere with the process of falling asleep, keeping the brain active even when the body is tired. Insomnia can exacerbate anxiety and depressive symptoms and anxiety and depression can further worsen insomnia, creating a vicious cycle. Insomnia, anxiety and depression may share some biological mechanisms, such as dysfunction in specific brain regions and neurotransmitter imbalance. Life stress, insufficient social support and other factors may also simultaneously affect the occurrence and development of insomnia, anxiety and depression. Moreover, when compared with depression, anxiety and sleep-related factors exhibited a stronger correlation. This finding aligns with the clinical observations reported here and research that patients with primary insomnia often experience anxiety related to their sleep patterns and that anxiety resulting from chronic insomnia is more prevalent than depression (Johnson et al., 2006; Stewart et al., 2006).

Finally, as a widely used analysis tool (Ballesio et al., 2016; Blanken et al., 2022; Hevey, 2018; Brohee et al., 2008), the network analysis employed in this study of the relationship between sleep quality and cognitive function offers a robust methodology. As demonstrated here, high sleep quality, sleep efficiency, immediate memory, and delayed memory underscore the significance of maintaining optimal episodic memory through high-quality and efficient sleep. Episodic memory, which encompasses the recollection of events, places and times, plays a pivotal role in daily life and learning. Inadequate or inefficient sleep may impede memory consolidation processes, thereby affecting the encoding and retrieval of new information. Furthermore, the correlation between sleep disturbances and daytime dysfunction with naming ability and language processing abilities suggests that disruptions in sleep patterns and daytime fatigue can hinder language processing skills. Proficient language abilities and vocabulary are essential for effective communication and the expression of ideas; however, sleep-related issues may compromise these capabilities, leading to challenges in daily communication and learning.

### Limitations

This study had several limitations. First, the sample size was relatively small, which may limit the generalizability of the findings. A larger sample size would typically provide more robust statistical power and a better representation of the broader population. However, this study serves as an initial exploration of the correlation between emotional symptoms, cognitive function changes, and the underlying network of sleep, emotional, and cognitive indicators in primary insomnia. The findings from this pilot study offer valuable insights and hypotheses that can guide future research. In our future studies, we plan to recruit a much larger and more diverse sample to validate and expand upon these preliminary results. This will help to enhance the generalizability of our findings and provide a more comprehensive understanding of the complex relationships involved. Second, the incorporation of objective measures such as biomarkers or physiological parameters would provide additional insights into the insomnia process. Further in-depth exploration of these mechanisms requires sophisticated research, especially at the molecular biology level. Finally, future research should focus in greater detail on interdisciplinary integration using advanced neuroimaging techniques, genetics and molecular biology methods to explore the mechanisms of insomnia and cognitive and emotional disorders. Simultaneously, individualized intervention measures should be developed and validated for both their long-term safety and effectiveness in different populations, including: Psychotherapy, pharmacotherapy, lifestyle adjustments and emerging digital therapies. Despite these limitations, this study contributes to the growing body of literature on primary insomnia and offers novel insights into cognitive dysfunction and emotional symptoms.

## Conclusion

This study revealed the complex interplay between insomnia and cognitive and emotional disorders, providing a new perspective and evidence for further understanding, diagnosis and treatment. In summary, this study not only deepens understanding of the mutual relationship between cognitive dysfunction and anxiety/depressive symptoms in sufferers of primary insomnia but also provides guidance for clinical practice, emphasizing the importance of comprehensive intervention. It also points to new directions for future research and lays a scientific foundation for improving the quality of life and health outcomes of patients.

## Data Availability

The raw data supporting the conclusions of this article will be made available by the authors, without undue reservation.
